# Clinical practice selectively follows acute appendicitis guidelines

**DOI:** 10.1007/s00068-022-02208-2

**Published:** 2023-01-31

**Authors:** Gary Alan Bass, Shahin Mohseni, Éanna J. Ryan, Maximilian Peter Forssten, Matti Tolonen, Yang Cao, Lewis J. Kaplan, ESTES SnapAppy Group, Rebecka Ahl Hulme, Alan Biloslavo, Hayato Kurihara, Isidro Martinez-Casas, Jorge Pereira, Arvid Pourlotfi, Nayef Louri, Yang Cao, Fatema Nedham, Thomas Noel Walsh, Jamal Hashem, Martin Corbally, Abeer Farhan, Hamad Al Hamad, Rawan Elhennawy, Mariam AlKooheji, Manar AlYusuf, Wissal Aknouche, Anas A. Zeidan, Yusuf S. Alsaffar, Edgar Lipping, Peep Talving, Sten Saar, Katrina Graumann, Liis Kibuspuu, Eduard Harkov, Gisele Aaltonen, Iines S. Sillman, Sami Haapanen, Hanna Lampela, Henna Sammalkorpi, Sofia Eskola, Altti Laakso, Johan Back, Ulla Kettunen, Antti M. Nummi, Anika Szwedyc, Taina Nykänen, Rolle Rantala, Elisa J. Mäkäräinen-Uhlbäck, Sanna A. Meriläinen, Heikki I. Huhta, Jukka M. J. Rintala, Kirsi E. M. Laitakari, Elina Lietzen, Paulina Salminen, Risto K. A. Rapola, Vahid Zangouri, Mohammad Y. Karami, Sedigheh Tahmasebi, Majid Akrami, Alireza Golchini, Faranak Bahrami, Sean M. Johnston, Sean T. Lim, Irele Ifijeh Ahonkhai, Eltahir Eltagani, Odhran K. Ryan, Ailbhe O’Driscoll-Collins, Aine O’Neill, Zakiya Penny, Orlaith Kelly, Carolyn Cullinane, Ian Reynolds, Helen Heneghan, Sean Martin, Des Winter, Matthew Davey, Maha Alkhattab, Aoif J. Lowery, Michael J. Kerin, Aisling M Hogan, Martin S Davey, Ke En Oh, Syed Mohammad Umar Kabir, Huilun Huan, Charlotte Aziz, Michael Sugrue, Jessica M. Ryan, Tara M. Connelly, Mohammad Alhazmi, Youssef Al-Mukhaizeem, Fiachra Cooke, Peter M. Neary, Arnold D. K. Hill, Michael R. Boland, Angus J. Lloyd, Frances Fallon, Eoin F. Cleere, James Toale, Patrick A. Boland, Michael Devine, Conor Keady, Sarah Hunter, M. Kevin Barry, Michael E. Kelly, Aidan T. O’Dowling, Ben Creavin, Dara O. Kavanagh, Paul Neary, Paul F F. Ridgway, Cathleen A. McCarrick, Jarlath Bolger, Barry Maguire, Cian Keogh, Surbhi Chawla, John Conneely, Emilie McCormack, Ben Shanahan, Nicola Raftery, Darragh Rice, Niall McInerney, Aine Stakelum, Jan Mares, Jonavan Tan, Mark Hanna, Ishwarya Balasubramanian, Christin Fleming, Guy Barsky, Gad Shaked, Simone Giudici, Martina Ceolin, Simona Mei, Francesca Mazzarella, Annalisa Zucca, Susanna Terranova, Nicolo de Manzini, Diego Visconti, Emanuele Doria, Mauro Santarelli, Giovanni Scotton, Francesca Notte, Giacomo Bertelli, Anna Malpaga, Giulia Armatura, Antonio Frena, Dario Tartaglia, Federico Coccolini, Camilla Cremonini, Enrico Cicuttin, Alessio Mazzoni, Massimo Chiarugi, Constança M. Azevedo, Filipa D. Mendes, Luis Q. Faria, Carlos Nazario, Daniela Machado, Miguel Semiao, Carlos Casimiro, Jose Pinto, Tiago Pavão, Raquel Pereira, Bruno Barbosa, Nadia Tenreiro, Catia Ferreira, Goncalo Guidi, Daniela C. Martins, Clara Leal, Bruno B. Vieira, Luís S. Castro, Aldara Faria, Alberto Figueira, Mauro Sousa, Pedro Rodrigues, Rodrigo Roquette, Ricardo Ribeiro, Paulo Cardoso, Joana Domingues, Maria Isabel Manso, Rute Pereira, Tatiana Revez, Bogdan D. Dumbrava, Florin Turcu, Ionut Hutopila, Bogdana Banescu, Gerald Filip, Catalin Copaescu, Marcos Alba Valmorisco, Isabel Manzano Martín, Rocio Martín García de Arboleya, José Ortega Seda, Pablo Rodríguez González, Jose Antonio Becerra Toro, Enrique Rodríguez Lara, Jose Antonio González Minchón, Juan José Segura-Sampedro, Sebastián Jerí-McFarlane, Alejandro Gil-Catalán, Andrea Craus-Miguel, Laura Fernández-Vega, Xavier González-Argenté, Mercedes Estaire-Gómez, Borja Camacho Fernández-Pacheco, Rebeca Vitón-Herrero, Elisa Jimenez-Higuera, Alejandro Barbero, José M. Valverde, Enrique Colás-Ruiz, Maria del Mar Escales-Oliver, Olga Claramonte-Bellmunt, Marta Castro-Suárez, Naila Pagés-Valle, José Andrés Cifuentes-Ródenas, Marta Merayo Alvarez, Jose Luis Michi Campos, Luis Alejandro García González, Beatriz Carrasco Aguilera, Jaime Iturbe Menéndez, Jose Luis Rodicio Miravalles, Carmen Rodríguez Haro, Sara Núñez O’Sullivan, Mariana García Virosta, María Hernández O’Reilly, Izaskun Balciscueta-Coltell, Javier Lorenzo-Perez, Sonia Martinez-Alcaide, Susana Martinez-Ramos, Maria Sebastian-Fuertes, Laura Gomez-Romer, Maria M. Pelloni, Aida Cristina Rahy-Martín, Andrés Felipe Yepes-Cano, Julio Reguera-Rosal, Jose A. Lopez-Ruiz, Beatriz Marenco, Marina Retamar-Gentil, Estela Romero-Vargas, Angeles Gil-Olarte, Aitor Landaluce-Olavarria, Begoña Estraviz-Mateos, Jose Mario De Francisco-Rios, Aitor Sainz-Lete, Ane Emaldi-Abasolo, Manolo Leon-Valarezo, Claudia C. Lopes Moreira, Aintzane Lizarazu Perez, Araceli Rodriguez Gonzalez, Iñigo Augusto Ponce, Ignacio Maria Goena Iglesias, Cristina González-Prado, Guillermo Cabriada, Beatriz López, Michelle C. Otero, Nerea Muñoz-Plaza, Alberto Palomo, Fernando Mendoza-Moreno, Manuel Díez-Alonso, Francisca García-Moreno-Nisa, Belén Matías-García, Enrique Ovejero-Merino, Ana Quiroga-Valcárcel, Luis Sánchez-Guillén, Inmaculada Oller-Navarro, Álvaro Soler-Silva, Antonio Francisco Sanchís-López, Francisco Blanco-Antona, Luis Muñoz-Bellvis, Jaime López-Sánchez, Sonsoles Garrosa-Muñoz, Beatriz Barón-Salvador, Juan Manuel Nieto-Arranz, Andrea Campos-Serra, Raquel Gràcia-Roman, Anna Muñoz-Campaña, Carla Zerpa-Martin, Andrea Torrecilla-Portoles, Tessa Landa, Virginia Durán Muñoz-Cruzado, Felipe Pareja-Ciuró, Daniel Aparicio-Sánchez, Eduardo Perea del Pozo, Sandra Dios-Barbeito, Carlos García-Sánchez, Antonio Jesús García-Moriana, Victor Turrado-Rodriguez, Roser Termes-Serra, Paula Gonzalez-Atienza, Xavier Morales-Sevillano, Alba Torroella, César Ginestà, Alfredo Escartín, Ferney Gomez, Ana Pinillos, Jaume Ortega, Guillermo Lopez, Eric Gutierrez, Estela Membrilla-Fernandez, Francisco Ocho-Segarra, Ana María González-Castillo, Amalia Pelegrina-Manzano, Juan Guzmán-Ahumada, Juan Jose Sancho-Insenser, María Lourdes García-Jiménez, Laura Castro-Diez, Manuel González-Bermúdez, Mónica Torres-Díaz, Carla Madarro Pena, Angélica Blanco Rodríguez, Dhanisha Trivedi, Souheil Reda, Hans Edvardsson, Lovisa Strömmer, Eva-Corina Caragounis, Karin Sillén, Sofia Warfvinge, Fredrik Bergstedt, Philip Enström, Harald Olsson, Anders Rosemar, Nathalie Young, Agnieszka Popowicz, Johanna Lerström, Johanna Jäderbo, Folke Hammarqvist, Hanna Zacharias, Maria B. Wikström, Anna Stene Hurtsén, Haytham Bayadsi, Emma Jansson, Nils Brunstrom, Ellen B. Malers, Per I. Loftås, Anders Möller, Elena Atanasova, Simone N. Zwicky, Beat Schnüriger, Olga Rutka, Arjun T. Kattakayam, Mushfique Alam, John V. Taylor, Andrei Mihailescu, Eszter T. Karip, Ehtisham Zeb, Adam O’Connor, Goran Pokusevski, Mansoor Khan, Charlotte Florance, Christie Swaminathan, Shameen Jaunoo, Mohammed Sajid, Caoimhe C. Duffy, John Rees, Mark J. Seamon, Niels D. Martin, Ian J. McCurry, Emily A. Vail, Bradford C. Bormann, Daniel C. Cullinane, Jaswin S. Sawhney, Jonathan Dreifus, Forest R. Sheppard, Raul Coimbra, Paul Albini, Sara Edwards

**Affiliations:** 1 Division of Traumatology, Surgical Critical Care & Emergency Surgery, Perelman School of Medicine at the University of Pennsylvania, Philadelphia, USA; 2 Division of Trauma & Emergency Surgery, Orebro University Hospital and School of Medical Sciences, Orebro University, Örebro, Sweden; 3 Center for Perioperative Outcomes Research and Transformation (CPORT), University of Pennsylvania, Philadelphia, USA; 4 Leonard Davis Institute of Health Economics (LDI), University of Pennsylvania, Philadelphia, USA; 5 Tallaght University Hospital, Dublin, Ireland; 6 Helsinki University Hospital HUS Meilahden Tornisairaala, Helsinki, Finland; 7 Department of Clinical Epidemiology and Biostatistics, School of Medical Sciences, Orebro University, Orebro, Sweden; 8 Corporal Michael Crescenz Veterans Affairs Medical Center, Philadelphia, USA

**Keywords:** Acute appendicitis, Observational cohort, Appendectomy, Guidelines

## Abstract

**Introduction:**

Acute appendicitis is a common surgical emergency, and the standard approach to diagnosis and management has been codified in several practice guidelines. Adherence to these guidelines provides insight into independent surgical practice patterns and institutional resource constraints as impediments to best practice. We explored data from the recent ESTES SnapAppy observational cohort study to determine guideline compliance in contemporary practice to identify opportunities to close evidence-to-practice gaps.

**Methods:**

We undertook a preplanned analysis of the ESTES SnapAppy observational cohort study, identifying, at a patient level, congruence with, or deviation from WSES Jerusalem guidelines for the diagnosis and management of acute appendicitis and the Surviving Sepsis Campaign in our cohort. Compliance was then correlated with the incidence of postoperative complications.

**Results:**

Four thousand six hundred and thirteen (4613) consecutive adult and adolescent patients with acute appendicitis were followed from date of admission (November 1, 2020, and May 28, 2021) for 90 days. Patient-level compliance with guideline elements allowed patients to be grouped into those with full compliance (all 5 elements: 13%), partial compliance (1–4 elements: 87%) or noncompliance (0 elements: 0.2%). We identified an excess postoperative complication rate in patients who received noncompliant and partially compliant care, compared with those who received fully guideline-compliant care (36% and 16%, versus 7.3%, *p* < 0.001).

**Conclusions:**

The observed diagnostic and treatment practices of the participating institutions displayed variability in compliance with key recommendations from existing guidelines. In general, practice was congruent with recommendations for preoperative antibiotic surgical site infection prophylaxis administration, time to surgery, and operative approach. However, there remains opportunities for improvement in the choice of diagnostic imaging modality, postoperative antibiotic stewardship to timely discontinue prophylactic antibiotics, and the implementation of ambulatory treatment pathways for uncomplicated appendicitis in the healthy young adult.

## Introduction

Acute appendicitis is one of the most prevalent general surgical emergencies worldwide [1–4]. The combination of resectional source control (appendectomy) and perioperative antimicrobial pharmacotherapy have served as the ‘gold standard’ treatment strategy for most with acute appendicitis [5]. However, decision-making for those with acute appendicitis has recently become more nuanced around treatment approach and timing, especially related to acute SARS-CoV-2 infection. Acknowledging this, the World Society of Emergency Surgery (WSES) identifies acute appendicitis as a global research priority in acute care surgery [6, 7].

Discouragement of the use of laparoscopy by guidelines from learned societies early in the COVID-19 pandemic accentuated a drift in ‘usual practice’ away from operative management for a variety of surgical conditions, including appendicitis [8, 9]. Unrelatedly, the observation that early appendicitis may be treated with antibiotics alone in selected patients had already prompted randomized controlled trial investigation [10–12]. However, the maturation of these trial results suggested poorer outcomes in nonoperative versus operative treatment of appendicitis [12–17]. Furthermore, as the pandemic progressed, and the virus mutated, it became apparent that the perioperative morbidity and mortality associated with acute SARS-CoV-2 infection appeared overstated [18–21]. Nonetheless, the influence of rapidly articulated professional organization guidance—ahead of evidence—and the rise of nonoperative management supported the growth of heterogenous patterns of practice for patients with acute appendicitis, arose that diverged from [22–26]

Consensus guidelines are often developed use the modified Delphi method, an iterative approach which originated in business management decision-making theory [22]. This approach leverages aggregated expert opinion anchored in data from previous scientific inquiry to harmonize clinical practice across healthcare settings [23]. The resulting guidelines, which exist for the diagnosis and treatment of many surgical diseases, are often sponsored by medical professional organizations, such as the European Society of Trauma and Emergency Surgery (ESTES), the Eastern Association for the Surgery of Trauma (EAST), the Society for Critical Care Medicine (SCCM), and the World Society of Emergency Surgery (WSES). Prompt initiation of empiric antimicrobial therapy and early definitive source control reduce the risk of infection-related complications including organ failure and mortality [24–26]. Antimicrobial stewardship, informed by the STOP-IT trial [27, 28], advocates for the discontinuation of antibiotics in a truncated time frame after achieving adequate source control [29]. These findings have been codified in several medical professional organization guidelines, such as the joint SCCM/European Society for Intensive Care Medicine (ESICM)-sponsored *Surviving Sepsis Campaign* (revised 2021) [30] and the WSES *Jerusalem Consensus Guidelines for the Diagnosis and Management of Acute Appendicitis* (revised 2020) [31].

In the SnapAppy study of usual care in appendicitis, we accrued a simultaneous multi-national observational sample of adult patients undergoing operation for acute appendicitis to determine current practice patterns. This non-interventional approach provides granular insights into current practice and how it embraces or deviates from published guidance. Since guideline adoption is generally nonuniform and may require decades to demonstrate widespread adoption, deviation is reasonably anticipatable [32, 33]. Furthermore, recognizing that individual clinicians direct bedside care within the context of institutional expertise and resources, we hypothesized that there would be broad divergence from established guidelines regarding antibiotic agent selection, antibiotic cessation timing, diagnostic modality selection, and operative approach in managing acute appendicitis.

## Methods

### Protocol

Using the ‘snapshot audit methodology,’ a validated prospective observational approach to studying epidemiology, treatment effectiveness and inter-institutional variations in practice patterns and guideline adherence [34], we constructed a defined dataset, in line with a pre-specified protocol registered with ClinicalTrials.gov (Trial # NCT04365491), which captures the contemporary epidemiology of appendicitis across 71 centers in 14 European, Middle Eastern, and North American countries (Bahrain, Estonia, Finland, Iran, Ireland, Israel, Italy, Portugal, Romania, Spain, Sweden, Switzerland, UK, and USA. We enrolled all consecutive adult patients (over 15 years of age) admitted with acute appendicitis in a 90-day window between November 1, 2020, and May 28, 2021, and followed those patients for 90 days post-admission (up to August 31, 2021). The study complied with the Strengthening the Reporting of Observational Studies in Epidemiology (STROBE) guidelines [35] and the Declaration of Helsinki [36].

### Center eligibility

To capture as broad a practice base as possible, any unit undertaking adult emergency general surgery was eligible to register patients in the study, unrestricted by minimum case volume, or center-specific limitations. The study protocol, and an invitation to participate, was disseminated to registered members of the European Society of Trauma and Emergency Surgery (ESTES), and through national surgical societies using societal email lists, social media announcements, announcement on the ESTES website, and through peer-to-peer word of mouth.

### Patient eligibility

All adult and adolescent patients (over 15 years of age) admitted for acute appendicitis were included in the current study. Acknowledging that there are several appendicitis grading systems, for simplicity and convenience, we used the AAST Anatomic Disease Severity grading system, which uses clinical, radiographic, operative, and pathologic criteria to assign an incrementing ordinal severity score of 1 (mild disease limited to the organ) to 5 (widespread severe disease) [37–40]. The American Society of Anesthesiologists (ASA) risk-stratification classification was reported. Patients who demonstrated mesenteric adenitis, or ovarian or colonic pathology were specifically excluded.

### Data capture

Data were recorded contemporaneously and stored on a secure, user-encrypted online platform (SMARTTrial^®^) without patient-identifiable information. Centers were asked to validate that all eligible patients during the study period had been entered and to attain > 95% completeness of data field entry prior to final submission. The database was closed for analysis on November 1, 2021. The SnapAppy protocol was designed so that usual patient follow-up pathways could be utilized to obtain outcomes data. No additional visits or changes to routine follow-up were made. However, local investigators were encouraged to be proactive in identifying post-diagnosis events (or lack thereof), within the limits of usual follow-up. These included reviewing the patient notes (paper and electronic) during admission and before discharge to note in-hospital complications, reviewing hospital systems to check for re-attendances or re-admissions, and reviewing postoperative radiology reports.

### Outcome measures

The primary outcome measures were documented adherence to practices recommended in the Surviving Sepsis Campaign 2021, and the WSES Jerusalem Guidelines 2020 for the diagnosis and management of acute appendicitis [30, 31]. Specifically, we focused on the following diagnostic and treatment guidelines that embrace antimicrobial stewardship: diagnosis by ultrasound or clinical examination in patients under the age of 40 years, and by CT in those over 40, the administration of first dose of preoperative antibiotics within 3 h of diagnosis, operative source control within 24 h of diagnosis, the omission of postoperative antibiotics in uncomplicated appendicitis, and the discontinuation of antibiotics within 3–5 days following adequate source control (Table 1). We tallied the five guideline elements described (Table 1), allowing patients to be grouped into those with full compliance (all 5 elements), partial compliance (1–4 elements), or noncompliance (0 elements). Secondary outcomes were inpatient length of stay and the incidence of postoperative complications (overall, and specific complications including surgical site infection [superficial, deep, organ space], superficial wound dehiscence, and postoperative ileus).

**Table 1:** Guideline compliance elements from the WSES Jerusalem Consensus Guidelines (2020) for the Diagnosis and Treatment of Acute Appendicitis, and the Surviving Sepsis Campaign Guidelines (2021) [30, 31]

Guideline domain	Recommendation/suggestion	Quality of evidence	Strength of recommendation
Diagnosis			
Clinical and Ultrasound	**WSES 1.7** We recommend the routine use of a combination of clinical parameters and US to improve diagnostic sensitivity and specificity and reduce the need for CT scan in the diagnosis of acute appendicitis	Moderate	Strong; 1B
Point-of-care Ultrasound	**WSES 1.10** We recommend POCUS as the most appropriate first-line diagnostic tool in both adults and children, if an imaging investigation is indicated based on clinical assessment	Moderate	Strong; 1B
CT	**WSES 1.9** We suggest that in high-risk patients younger than 40 years old (with AIR score 9–12 and Alvarado score 9–10 and AAS ≥ 16), CT may be avoided before proceeding to diagnostic + / − therapeutic laparoscopy	Moderate	Weak; 2B
CT	**WSES 1.12** We recommend cross-sectional imaging before surgery for patients with normal investigations but non-resolving right iliac fossa pain, and those over the age of 40 years. After negative imaging, initial nonoperative treatment is appropriate. However, in patients with progressive or persistent pain, explorative laparoscopy is recommended to establish/exclude the diagnosis of acute appendicitis or alternative diagnoses	High	Strong; 1A
Surgical treatment			
Source control time to OR	**WSES 3.1** We recommend planning laparoscopic appendectomy for the next available operating list within 24 h in case of uncomplicated acute appendicitis, minimizing the delay wherever possible	Moderate	Strong; 1B
Source control time to OR	**WSES 3.2** We recommend against delaying appendectomy for acute appendicitis of any grade needing surgery beyond 24 h from the admission	Moderate	Strong; 1B
Antimicrobial stewardship			
Time to first-dose antibiotics	**SSC 14** For adults with possible sepsis without shock, we suggest a time-limited course of rapid investigation and if concern for infection persists, the administration of antimicrobials within 3 h from the time when sepsis was first recognized	Weak	Very low
Preoperative antibiotics	**WSES 7.1** We recommend a single preoperative dose of broad-spectrum antibiotics in patients with acute appendicitis undergoing appendectomy	High	Strong; 1A
Duration of postoperative antibiotics	**WSES 7.1** We recommend against postoperative antibiotics for patients with uncomplicated appendicitis	High	Strong; 1A
Duration of postoperative antibiotics	**WSES 7.2** We recommend against prolonging antibiotics longer than 3–5 days postoperatively in case of complicated appendicitis with adequate source-control	*High*	*Strong; 1A*

### Statistical analysis

All descriptive analyses were conducted with the statistical software the jamovi project (2022). *jamovi*. (version 2.3), [Computer Software, Retrieved from https://www.jamovi.org.] running the *gtsummary, ClinicoPath* and *ggstatsplot* packages. Normally distributed variables were presented as means and standard deviations (SDs), while the median and interquartile range (IQR) were used for non-normally distributed variables. Categorical variables were summarized as counts and percentages.

*Ethical considerations.*

All participating centers secured IRB approval or equivalent. No patient consent was sought since the current study was purely observational. All data were de-identified when uploaded to the secure study database (SMARTTrial^®^), in compliance with HIPAA and EU GDPR legislation.

## Results

### Patient demographics

Four thousand six hundred and thirteen (4613) consecutive adult patients with acute appendicitis were followed from date of admission (November 1, 2020, and May 28, 2021) for 90 days. Their median age was 36 years (IQR 25–51), and there was a slight male preponderance (55.2%). Patients had a mean body mass index of 26.5 (± 12.1). Most patients were risk stratified as low risk to undergo general anesthesia by the American Society of Anesthesiologists (ASA) classification (ASA ≤ 2: 90.4%). Approximately 2% of patients had an active COVID-19 infection on admission, with a further 2.6% reporting prior infection.

### Diagnostic modality

The diagnosis of acute appendicitis was made on clinical findings in 512 (11.1%) patients, combining clinical findings and transabdominal ultrasound (either at point of care or in the radiology department) in 1436 (31.3%) and with axial imaging (CT) in 2644 (57.6%). Ninety percent of patients over the age of 40 underwent diagnostic CT.

### Source control

Of the 4,613 patients diagnosed with acute appendicitis who underwent a source control intervention, 4,391 (95.2%) underwent appendectomy, with the vast majority being operated upon within 24 h (87.4%). Interventional radiological drainage without operation was undertaken in 1.2% of cases. Most appendectomies were performed laparoscopically (85.8%). The conversion rate was approximately 3%, and the incidence of primary open surgery was 9.9%. Frank pus was observed in 16.8% of cases at operation. Most appendix specimens were submitted for histopathological evaluation (97.4%); 1.4% of patients had some form of appendiceal neoplasm upon histopathological evaluation.

### Perioperative antimicrobial stewardship

While almost all patients appropriately received preoperative antibiotic therapy (96.6%), the SSC target of a first dose within 3 h for those with confirmed or highly suspected sepsis was achieved in just 42.3% of patients. The most commonly prescribed antimicrobials were amoxicillin/clavulanic acid, or a cephalosporin with or without metronidazole (77.5%); other local variations were noted.

While data on postoperative antibiotic prescription are missing for 366 (8.3%) patients who underwent appendectomy, 62.5% of patients received postoperative antibiotics. Postoperative antibiotics were continued in 1,589 (49.7%) patients with Grade I (uncomplicated) appendicitis and 578 (71.4%) of patients with Grade II (gangrenous, non-perforated) appendicitis (see Table 2). The mean (SD; range) duration of postoperative antibiotics in Grade I and Grade II appendicitis was found to be 3.7 (4.1; 0–50) days and 4.3 (3.7; 0–21) days, respectively.

**Table 2:** Select guideline compliance metrics—grouped by AAST disease severity grade and overall patients

Characteristic	Grade I (*N* = 3203)	Grade II (*N* = 809)	Grade III (*N* = 277)	Grade IV (*N* = 288)	Grade V (*N* = 36)	All Patients (*N* = 4613)	*p* value
Method of diagnosis							< 0.001^b^
CT	1929 (60.5%)	157 (19.4%)	253 (92%)	275 (95.8%)	30 (83.3%)	2644 (57.6%)	
Over 40	929 (88.3%)	105 (84.0%)	186 (94.4%)	204 (97.6%)	20 (90.9%)	1444 (90.0%)	
Ultrasound	951 (29.8%)	453 (56.1%)	19 (6.9%)	9 (3.1%)	4 (11.1%)	1436 (31.3%)	
Clinical	306 (9.6%)	198 (24.5%)	3 (1.1%)	3 (1%)	2 (5.6%)	512 (11.1%)	
Missing	17	1	2	1	0	21	
Time to first-dose antibiotics^a^							
< 1 h	359 (11.8%)	102 (13%)	33 (12%)	43 (15.9%)	8 (22.9%)	545 (12.4%)	
1–3 h	817 (26.9%)	229 (29.2%)	91 (33.1%)	83 (30.6%)	13 (37.1%)	1233 (28%)	
3–6 h	796 (26.2%)	248 (31.7%)	80 (29.1%)	77 (28.4%)	8 (22.9%)	1209 (27.5%)	
> 6 h	1061 (35%)	204 (26.1%)	71 (25.8%)	68 (25.1%)	6 (17.1%)	1410 (32.1%)	
Missing	170	26	2	17	1	216	
First-dose antibiotics within 3 h	1346 (42%)	357 (44.1%)	126 (45.5%)	143 (49.7%)	22 (61.1%)	1994 (43.2%)	0.015^b^
Time to OR^a^							
< 6 h	555 (17.9%)	132 (16.8%)	58 (21.7%)	39 (18.5%)	12 (34.3%)	796 (18.1%)	
6–12 h	1109 (35.8%)	285 (36.3%)	102 (38.2%)	49 (23.2%)	11 (31.4%)	1556 (35.4%)	
12–24 h	1062 (34.3%)	279 (35.5%)	80 (30%)	72 (34.1%)	7 (20%)	1500 (34.1%)	
> 24 h	369 (11.9%)	89 (11.3%)	27 (10.1%)	51 (24.2%)	5 (14.3%)	541 (12.3%)	
Missing	108	24	10	77	1	220	
Preoperative antibiotics	3092 (96.6%)	787 (97.3%)	271 (97.8%)	268 (93.4%)	36 (100%)	4454 (96.6%)	0.013^b^
Missing	1	0	0	1	0	2	
Postoperative antibiotics	1589 (49.7%)	578 (71.4%)	263 (94.9%)	279 (97.6%)	36 (100%)	2745 (59.6%)	< 0.001^b^
Missing	4	0	0	2	0	6	
Postoperative antibiotic duration^~^	3.7 (4.1; 0–50)	4.3 (3.7; 0—21)	8.5 (4.2; 0–21)	10.5 (5.3; 0–33)	11 (5; 1–21)	4.6 (4.6; 0–50)	< 0.001^c^
Missing	474	83	13	15	3	588^d^	

^a^Time from diagnosis (hours); ^~^mean days (SD; range)

^b^Pearson's Chi-squared test

^c^Linear model ANOVA

^d^Includes 222 patients who did not undergo surgery; thus the true missing figure is 366

### Composite measure of compliance and postoperative complications

We tallied patient-level compliance with the five guideline elements described above (Table 1) allowing patients to be grouped into those with full compliance (all 5 elements: 13%), partial compliance (1–4 elements: 87%) or noncompliance (0 elements: 0.2%). We identified an excess postoperative complication rate in patients who received noncompliant and partially compliant care, compared with those who received fully guideline-compliant care (36% and 16%, versus 7.3%, *p* < 0.001). While the Clavien–Dindo 30-day complication classification and the absolute numbers for individual infective complications (wound infection, postoperative ileus, etc.) are tabulated in Table 3, the small event rate means that statistically significant differences are unlikely to have clinical significance. Country-level comparisons in compliance rates are tabulated in Table 4, with full compliance ranging from 0 to 45% (mean = 13%, SD = 14%). Greatest rates of full compliance were seen in patients treated in Romania, USA, and Estonia. Partial compliance was more common, ranging between 55 and 100%.

**Table 3:** Crude complication rate, grouped by full (5/5), partial (1–4/5) or noncompliance (0/5) with selected guidelines

Characteristic	*N*	Fully compliant *N* = 588 (13%)	Partially compliant *N* = 4014 (87%)	Noncompliant *N* = 11 (0.2%)	*p*-value
Any complication	4,610	43 (7.3%)	624 (16%)	4 (36%)	< 0.001
Wound infection	4,604	4 (0.7%)	81 (2%)	2 (18%)	0.002
Wound dehiscence	4,604	4 (0.7%)	26 (0.6%)	0 (0%)	0.80
Pelvic abscess	4,604	3 (0.5%)	143 (3.6%)	0 (0%)	< 0.001
Subphrenic abscess	4,604	0 (0%)	8 (0.2%)	0 (0%)	0.61
Postoperative sepsis	4,604	0 (0%)	33 (0.8%)	0 (0%)	0.050
Postoperative ileus	4,604	3 (0.5%)	117 (2.9%)	0 (0%)	< 0.001
Clavien–Dindo 30-day complications	4,415				< 0.001
1		11 (1.9%)	156 (4.1%)	1 (10%)	
2		8 (1.4%)	170 (4.4%)	2 (20%)	
3a		1 (0.2%)	68 (1.8%)	0 (0%)	
3b		1 (0.2%)	48 (1.3%)	0 (0%)	
4a		0 (0%)	2 (< 0.1%)	0 (0%)	
4b		0 (0%)	1 (< 0.1%)	0 (0%)	
5		0 (0%)	7 (0.2%)	0 (0%)	
None		545 (96%)	3,387 (88%)	7 (70%)	
Missing		22	175	1	

Selected guidelines were: preoperative antibiotic administration, first-dose antibiotics within 3 h of diagnosis (Surviving Sepsis Campaign 2021), time to OR less than 24 h, omission of postoperative antibiotics in uncomplicated appendicitis, and diagnosis using clinical examination or ultrasound in patients under 40 years of age, or using CT in patients over 40 years

**Table 4:** Full (5/5), partial (1–4/5) or noncompliance (0/5) with selected guidelines, divided by country

	Fully compliant		Partially compliant		Noncompliant		Total
Country	*n*	%	*n*	%	*n*	%	*n*
Bahrain	10	5%	211	95%	1	0%	222
Estonia	54	27%	147	73%	0	0%	201
Finland	47	7%	594	93%	0	0%	641
Iran	0	0%	88	100%	0	0%	88
Ireland	27	6%	418	94%	1	0%	446
Israel	0	0%	28	100%	0	0%	28
Italy	7	6%	113	94%	0	0%	120
Portugal	8	5%	161	95%	0	0%	169
Romania	9	45%	11	55%	0	0%	20
Spain	197	17%	962	83%	0	0%	1159
Sweden	112	11%	947	89%	0	0%	1059
Switzerland	2	7%	26	93%	0	0%	28
UK	10	6%	139	88%	9	6%	158
USA	105	38%	169	62%	0	0%	274
Overall	588	13%	4014	87%	11	0%	4613

Selected guidelines were: preoperative antibiotic administration, first-dose antibiotics within 3 h of diagnosis (Surviving Sepsis Campaign 2021), time to OR less than 24 h, omission of postoperative antibiotics in uncomplicated appendicitis, and diagnosis using clinical examination or ultrasound in patients under 40 years of age, or using CT in patients over 40 years

### Outcomes for appendicitis patients

Appendicitis patients had a median length of hospital stay of 2 days. Fewer than 1% of patients required ICU care. A postoperative complication was suffered by 17.3% of patients within 30 days of surgery, with the most common being the development of a pelvic abscess (3.2%), ileus (2.6%), or a surgical site infection (1.9%). However, only 2.8% of all patients suffered a severe complication (Clavien–Dindo classification ≥ 3a), while 1.6% required reoperation. A total of 7 patients (0.2%) died within the first month after surgery. Median (IQR) length of hospital stay in days was significantly shorter in patients who received fully compliant care (1.2, 0.8–1.7 days), compared with those who received partially compliant (2.0, 1.4–3.6 days) or noncompliant care (4.3, 3.2–8.1 days) [*p* < 0.001].

## Discussion

The SnapAppy analysis of the current state of acute appendicitis management offers valuable insights regarding practice patterns and outcomes across widely disparate health systems and settings. Besides affording comparisons between different countries, these data allow us to explore how current practice interfaces with existing evidence regarding that care. To that end, we have compared observational data with two major guidelines—that of the World Society of Emergency Surgery for the management of patients with acute appendicitis and that of the Surviving Sepsis Campaign for the management of patients with sepsis or septic shock (Table 1) [30, 31]. While the SSC guidelines may be viewed as primarily appropriate for medical disease, or postoperative infection such as pneumonia, recent iterations of the guidelines also highlight the importance of source control for patients with a source controllable lesion [30]. Therefore, appendectomy for acute appendicitis provides an ideal opportunity to achieve rapid and effective source control in the absence of perforation.

Guidelines provide an evidence base upon which clinicians may rely to inform care decisions [41]. Often, guidelines are generated using a modified Delphi consensus approach that yields recommendations, suggestions, best practices, and the recognition that data are sufficiently lacking for some topics to preclude guidance [22, 23]. Some guidelines, but not all, are accompanied by a ‘bundle’ that provides an implementation strategy that translates to the bedside [41]. When there is no offered implementation strategy, the individual clinician, group, or facility must devise how best to incorporate the evidence base into their practice. Unsurprisingly, the completeness with which evidence is embraced within practice is often less than uniform, creating what has been termed an ‘evidence-to-practice’ gap [42]. The abrogation of such gaps across all disciplines is a key priority of the expanding field of implementation science. The evidence-to-practice gap bears important implications for care quality, safety, and cost, rendering it imperative to understand what supports the creation of the gap.

Established drivers of the evidence-to-practice gap include individual, environment, and system factors. Established practice is generally comfortable for the clinician, and inertia may impede change. The notion that trial patients from whom the data are derived are different from those the clinician treats has some validity, especially when the data flow from Randomized Clinical Trials with restrictive entry criteria. However, a host of other assessments, like this study, is not restricted and provides evidence that may be broadly applied. Other clinician factors include a lack of awareness of newly published evidence, especially if the data are published behind a pay wall. Guidelines, on the other hand, are often freely accessible, even if only as an executive summary. Additional impediments are those related to the environment of care and the healthcare system. The lack of an electronic health record (EHR), as is the case in many of our contributing centers, may confound obtaining longitudinal data for assessment. Practice within a resource limited space, including those within low- and middle-income countries (LMIC), may constrain diagnostic modality availability, as well as therapeutic agent selection. Patients operated after 24 h may reflect lack of OR availability in resource constrained settings, a constraint that would not be remedied by an Emergency General Surgery service—an initiative that durably decreased time to OR for time sensitive conditions [43, 44]. Clinician availability may reduce the oversight required for timely termination of perioperative antibiotics, in the absence of an established pathway, or advanced practice provider (APP) to help guide care. These aspects likely contributed to the low frequency of an enhanced recovery after surgery (ERAS) approach that includes same day discharge for uncomplicated appendicitis in otherwise healthy adults [45, 46].

Nonetheless, our data demonstrate that practice can conform to guideline-based recommendations—which suggests the lack of an evidence-to-practice gap—across very different healthcare systems (USA, Estonia, Romania), at least in participating centers. Despite the ability to embrace guideline recommendations in select sites, most centers demonstrated failures in guideline adoption. These failures were most notable in antibiotic cessation timing and inpatient care (time to operation, and the lack of outpatient care for uncomplicated appendicitis). While not readily apparent on an individual patient basis, collectively, these choices exert major impacts on cost and serve as a driving force for the genesis of multidrug-resistant organisms [47–49]. Individual clinician management approaches influence microbial ecology within the hospital and the community, including chronic care facilities. Accordingly, antimicrobial stewardship programs (ASPs) have arisen to help guide appropriate antibiotic selection and cessation. ASPs bring additional clinicians onto the team to support embracing new evidence and incorporating it into practice but are insufficient in isolation. Other approaches are warranted to systematically close the gap.

A variety of methods may help close the evidence-to-practice gap and are presented below in order of increasing difficulty but also increasing anticipated efficacy. Clinician education is straightforward and may use a variety of platforms from traditional didactics to ‘just-in-time’ digital platform-based training. Despite the ease of providing education, it is challenging to demonstrate robust practice change in its wake [50–52]. Providing an incentive to change practice is more appealing, especially when financially based, but requires institutional resources or insurer resources to realize; incentives may be especially problematic in resource limited spaces. Exacting a penalty for compliance failure may be effective but then requires data reporting, analysis, and a larger system to ensure accurate attribution and penalty application. This is especially true when the penalty is related to finances that flow to an individual clinician as opposed to an established institution. Public reporting of compliance and outcomes may be built as an outgrowth of data acquisition and works best when it is mandated by a state or national agency. Such is the case for the New York State Department of Health’s mandatory reporting around sepsis care. There is substantial pressure for institutional performance when it is documented as lagging behind that of other institutions or practitioners. Finally, establishing a local champion who can access clinician specific data, review it with them, and provide peer-to-peer education and feedback holds the potential to be quite effective [53–55]. However, that individual must be credible, have sufficient time to do so, be compensated for time and effort, and work within a medical staff that is willing to have their practice examined by a peer. In the private sector, that peer may also compete for the same patients, relegating such ‘counselling’ to teaching institutions where such competition is less applicable. When such a system is feasible, the peer reviewer functions as a team member for the clinician, a post that may be particularly important for those in solitary practice.

Our observational study of acute appendicitis management offers a view of current practice while also demonstrating important limitations. First, due to time-bound simultaneous patient accrual, our data do not capture outcomes beyond 90 days after admission. Second, the original intention of the 90-day follow-up informed by anecdotal experience during the early phase of the COVID-19 pandemic was to capture early readmission with recrudescent appendicitis due to failure of nonoperative antibiotic therapy. However, very few patients undergoing nonoperative therapy were captured in our dataset. This is at least in part be explained using operating room logs to identify enrollable patients at centers without an EHR. Third, granular elements of patient care were not captured including, but not limited to, anesthetic technique, culture data, and specific antibiotic prescription. However, those elements were not the focus of the study. Furthermore, due to the heterogeneity on a national level, socioeconomic status, race/ethnicity, and insurance status were not recorded. Fourth, the correlations presented are merely associative and should not be construed as indicating causation, especially regarding complications that were quite infrequent. Prospective correlative analysis of outcomes would require a population-level epidemiologic study. Fifth, we did not assess all the bundle elements from the SSC as that inquiry would require a granularity that exceeded the scope of this investigation. Finally, as these data were limited to adult patients admitted for appendicitis, they should not be extrapolated to the pediatric population.

## Conclusions

The observed diagnostic and treatment practices of the participating institutions displayed variability in compliance with key recommendations from existing guidelines (2020 WSES Jerusalem and the 2021 Surviving Sepsis Campaign) [30, 31]. In general, practice was congruent with recommendations pertaining to preoperative antibiotic surgical site infection prophylaxis administration, time to surgery, and operative approach. However, there remains opportunities for improvement in the choice of diagnostic imaging modality, postoperative antibiotic stewardship to timely discontinue prophylactic antibiotics, and the implementation of ambulatory treatment pathways for uncomplicated appendicitis in the healthy young adult. Data from the SnapAppy are hypothesis generating and should optimally inform future investigations and implementation science initiatives specifically designed to close the evidence-to-practice gap.

## Data Availability

The ESTES SnapAppy Group welcomes the use of these de-identified pooled data for further research that benefits patients. Requests can be submitted to the ESTES Research Committee. Release is subject to their approval and the appropriate safeguarding as determined by applicable legislation (GDPR and HIPAA).
